# The course of pain and function in osteoarthritis and timing of arthroplasty: the CHECK cohort

**DOI:** 10.1080/17453674.2018.1502533

**Published:** 2018-10-23

**Authors:** Maaike G J Gademan, Hein Putter, Wilbert B Van Den Hout, Margreet Kloppenburg, Stefanie N Hofstede, Suzanne C Cannegieter, Rob G H H Nelissen, Perla J Marang–Van De Mheen

**Affiliations:** a Department of Orthopaedics, Leiden University Medical Center;; b Department of Clinical Epidemiology, Leiden University Medical Center;;; c Department of Biomedical Data Sciences;, Leiden University Medical Center;; d Department of Rheumatology, Leiden University Medical Center, Leiden, The Netherlands

## Abstract

**Background and purpose —** It is unknown whether different trajectories of pain or function are associated with timing of total hip or knee arthroplasty (THA/TKA) in osteoarthritis (OA) patients. We investigated this association in early symptomatic OA patients.

**Patients and methods —** Data from the prospective Dutch CHECK cohort (patients with early hip/knee OA complaints) covering 9 years of follow-up were used. Pain and function were measured annually using the WOMAC questionnaires. Changes in pain/function over time were estimated using a linear mixed model adjusted for baseline age, sex, BMI, maximal Kellgren and Lawrence score, number of painful joints, and comorbidities. The same covariates were included in a Cox regression model, with time to first arthroplasty as event. Both were combined in a joint model to assess the association between changes in pain/function and time to arthroplasty.

**Results —** Of the 868 eligible patients, 84 received a TKA/THA during follow-up. Patients receiving arthroplasty were somewhat older, had a higher Kellgren and Lawrence score and worse WOMAC scores at baseline. Irrespective of receiving arthroplasty, about two-thirds of the patients showed at least 1 period of deterioration of pain/function (≥ 10 points WOMAC subscale). In approximately two fifths this deterioration was followed by another deterioration in the following year. Worse pain and function levels increased the hazard of receiving THA/TKA (1.08 [95% CI 1.06–1.10] for pain and 1.07 [CI 1.05–1.08] for function). Changes in pain or function over time were not associated with timing of THA/TKA

**Interpretation —** Worse pain and function levels rather than long-term changes are associated with timing of THA/TKA.

Although total knee arthroplasty (TKA) and total hip arthroplasty (THA) are effective interventions, their optimal timing is unknown. This is illustrated by the varying disease severity when surgery is performed across centres in Europe and Australia (Ackerman et al. 2009, Dieppe et al. 2009). Indication criteria for TKA/THA in guidelines acknowledge pain, function, radiological changes, and insufficient effect of non-operative therapy (Jordan et al. 2003, Zhang et al. 2005, 2008, Gademan et al. 2016). Therefore the first step towards knowledge on optimal timing of TKA/THA is to investigate the time course of pain, function, and joint degeneration before surgery.

Several studies have shown that pain and function in knee osteoarthritis (OA) are persistent rather than progressive (Leffondre et al. 2004, Yusuf et al. 2011, Pisters et al. 2012, Collins et al. 2014, Wesseling et al. 2015). For example, in our Cohort Hip and Cohort Knee (CHECK) consisting of patients with pain and/or stiffness of knees and/or hips we showed previously that 3 stable pain trajectories were found with marginal, mild, and moderate pain (Wesseling et al. 2015). Similar results have been shown for function (Leffondre et al. 2004, Yusuf et al. 2011, Pisters et al. 2012, Riddle et al. 2013, Collins et al. 2014, Wesseling et al. 2015). If pain and function in OA are indeed stable and not progressive, optimal timing of THA/TKA would depend only on the prosthesis life span because the gain after surgery will be equal irrespective of timing. However, if the condition is progressive, one would expect TKA/THA patients to experience increased pain and declining function, particularly in the years before arthroplasty. Here gain after surgery may be less if preoperative pain/function is worse (Hofstede et al. 2016); patients with worse preoperative pain/function tend to have worse outcomes although they improve more than patients with fewer preoperative complaints. Only 1 previous study investigated trajectories of pain and function before arthroplasty and showed that mean pain increased and function decreased during the last 2.5 years before surgery (Riddle et al. 2013). However, there was no control group, so it is possible that patients not receiving arthroplasty reported similar changes in pain/function.

Therefore, we assessed whether different trajectories of pain or function are associated with timing of THA/TKA. To answer this question we combined the repeated measurements of pain and function among early symptomatic OA patients over 9 years, with a time-to-event analysis.

## Patients and methods

### Study type and setting

This is a nationwide Dutch observational prospective cohort study conducted in cooperation with 10 general and academic hospitals located in urbanized and semi-urbanized regions.

### Population

A detailed description of the CHECK cohort has been reported elsewhere (Wesseling et al. 2014). Eligible patients were recruited between 2002 and 2005 and had knee/hip pain, were between 45 and 65 years of age and within 6 months of their first GP visit for these complaints. There were no radiographic signs required to take part in the cohort. Exclusion criteria were: any other pathological condition than OA explaining the symptoms, comorbidity precluding physical evaluation or follow-up of 10 years, malignancy in the past 5 years, and inability to understand Dutch. We included 1,002 patients. After inclusion, patients were divided into 2 groups: patients with relatively more severe symptoms (n = 861, Figure 1, see Supplementary data) visited the research centre each year for collection of clinical, radiological, and biochemical data; patients with mild symptoms (n = 141) visited the research centre at years 0, 2, 5, 8, and 10. Patients could shift to the more serious symptoms group, and were then measured annually as well (n = 79 after 5 years) (Wesseling et al. 2014).

### Measurements

#### Demographics

At baseline patients reported BMI and the number of self-reported comorbidities according to the Statistics Netherlands questionnaire (Botterweck et al. 2001). Self-reported stiffness was assessed at baseline using the Western Ontario and McMaster Universities Osteoarthritis Index (WOMAC) stiffness subscale (Bellamy et al. 1988). At baseline, 2, 5, and 8 years knee radiographs were taken in a weight-bearing semi-flexed posteroanterior view and for the hip in weight-bearing anteroposterior radiographs of the pelvis with hips in 15° internal rotation. Both knee and hip radiographs were read by observers blinded to all patient characteristics and the radiographs were scored according to Kellgren and Lawrence (K&L). In 38 participants radiographs were blindly scored by 5 trained observers (4 research assistants and 1 experienced general practitioner reader) in a paired fashion, with known sequence and interobserver variability was tested (Cohen’s kappa =0.60 for K&L ≥ 2 in knees at 5-year follow-up). The Cohen’s kappa is rather low because of the relatively low frequency of radiographic abnormalities. Therefore a prevalence-adjusted bias-adjusted kappa (PABAK) score was assessed. The PABAK score for reliability on progression of OA (KL score) in the knee from 0 to 5 years was 0.82, with a 90% average agreement. Similar results were found for the hip (Damen et al. 2014).

#### Pain and function

At baseline, pain history was assessed by a rheumatologist. Knee and hip pain were classified as present or absent and the total number of painful joints was calculated. Self-reported pain and function were assessed at each visit using the WOMAC pain/function subscales, each scored on a 5-point Likert scale (0 = no pain/good function (Bellamy et al. 1988). The pain and function subscales comprise respectively 5 and 17 questions.

#### Arthroplasty

During each visit patients were asked whether they had received a TKA/THA. If so, the date of surgery was noted.

### Specific methods current study

We included all measurements until 9 years of follow-up. Only patients with at least 2 measurements and no missing values for baseline characteristics were included (sex, age, BMI, maximal K&L, hip/knee joint pain, and comorbidities) (n = 868, i.e., 87%). The median follow-up was 9.0 (IQR 8.9–9.1) years. We used questionnaire data, data derived by a clinician from medical records, and radiographical data (Figure 1, see Supplementary data). Pain and function were assessed by the standardised WOMAC pain score ((total pain score) × (100/20)) and function score ((total function score) × (100/68)). To investigate whether THA/TKA patients more often experienced episodes of deteriorating pain or function prior to THA/TKA, compared with patients not receiving arthroplasty, we defined deteriorating pain or function as the first ≥10 points increase on the standardised WOMAC pain/function subscales compared with the year before. Further deterioration in the year thereafter was defined as any increase in WOMAC pain/function. When patients received multiple arthroplasties during follow-up, the first arthroplasty was taken as event, and their follow-up was censored. Baseline maximal K&L was defined as the highest K&L in the hip/knee joints at baseline. Maximal K&L during follow-up was defined as the highest K&L in the hip/knee in all measurements.

### Statistics

Differences in baseline characteristics between patients with and without a TKA/THA during follow-up were investigated with Student’s t-test (continuous outcomes) or a chi-square test (categorical outcomes). To depict the timing of arthroplasties, a Kaplan–Meier curve was estimated.

Descriptive statistics of the episodes of deterioration were reported, e.g., proportion of patients with deterioration of pain/function among those with and without arthroplasty. Differences between groups were evaluated with logistic regression adjusted for time to follow-up.

In addition we fitted linear mixed-effects models with a random slope and random intercept per patient to the reported levels of pain and function. The random-effects part describes the time course of pain and function for each patient and takes into account the within-subject correlation of different measurements. Within these models we adjusted for age, sex, BMI, maximal K&L, number of painful joints, and comorbidities at baseline. As such we could estimate the adjusted course of pain and function for each patient separately. Pain and function are not assumed to be constant between successive measurements, so that direct inclusion in a time-dependent Cox model may produce biased results. Therefore, we included the estimated courses of pain from the linear mixed-effect model (each point in time can be estimated with this model) as a time-dependent covariate in the Cox model. The latter was done by joint modelling the longitudinal and survival data with the JM package (1.4-2) (R version 3.2.3) (Rizopoulos 2010) (Supplementary file). We used a piecewise constant baseline hazard. In the Cox model we adjusted for age, sex, BMI, maximal K&L, number of painful joints, and comorbidities at baseline.

### Sensitivity analysis

We adjusted for maximal K&L during follow-up instead of baseline maximal K&L, as radiographic joint deterioration is an indication for THA/TKA. Second, we checked whether adding the slope of the linear mixed model to the joint model changed our results. As the slope refers to the long-term change over time in pain/function during 9 years of follow-up, we assessed whether these were associated with the likelihood of receiving arthroplasty.

### Ethics, funding, and potential conflicts of interest

The medical ethics committees of all participating centres approved the study and all patients gave written informed consent. This study was funded by CHECK and by a separate grant (Dutch Arthritis Foundation (ARGON, BP12-3-401)). This foundation did not play a role in the study’s design, conduct, or reporting. The authors have no conflicts of interest to declare.

## Results

Of the 868 included patients, 84 received TKA (n = 29) or THA (n = 55) during 9 years of follow-up. Patients receiving an arthroplasty were somewhat older at baseline, had higher K&L scores, and worse WOMAC scores compared with patients without arthroplasty (Table 1). None of the patients had a maximal K&L score ≥2. Compared with patients not included (n = 134), our population had similar baseline characteristics, with the exception of better WOMAC scores (Table 2, see Supplementary data). Of the 84 arthroplasties, 67 were implanted in the first 5 years (Figure 2, see Supplementary data). Mean WOMAC scores before arthroplasty were respectively 43.5 (SD 20.6) for function and 45.2 (19.2) for pain.

**Table 1.** Baseline patient characteristics of patients who received total joint replacement somewhere during follow-up and the patients who did not

	Total joint replacement		
	No n = 784	Yes n = 84	p-value
Age (years)	56 (5.3)	58 (4.3)	0.001
Sex:			
Male	161 (21)	18 (21)	
Female	623 (80)	66 (79)	
BMI	26 (4.1)	27 (4.6)	0.4
Comorbidities	1.9 (1.5)	1.7 (1.5)	0.3
Number of painful joints:			
1	230 (29)	32 (38)	
2	338 (43)	32 (38)	
3	114 (15)	11 (13)	
4	102 (13)	9 (11)	0.4
Maximal Kellgren and Lawrence score:			
0	291 (37)	11 (13)	
1	493 (63)	73 (87)	< 0.001
WOMAC standardized subscales:			
Pain	24 (16)	33 (19)	< 0.001
Function	22 (17)	32 (17)	< 0.001
Stiffness	32 (21)	38 (20)	< 0.01

WOMAC: Western Ontario and McMaster Universities Osteoarthritis Index. Continuous variables are shown as mean (SD), categorical variables are shown as number (percentage)

### Episodes of deterioration of pain and function

There was no difference in the percentage of patients that showed at least 1 period of deterioration of pain between patients who did or did not receive TKA/THA during follow-up: 56/85 patients versus 531/784. 13 of these 56 TKA/THA patients showed further deterioration of pain in the year thereafter against 88 of the 531 patients without arthroplasty.

Similar results were found for function; at least 1 period of deterioration of function before arthroplasty was found in 56 of 84 TKA/THA patients and in 540 of 784 patients without arthroplasty. This deterioration was followed by further deterioration in the following year in 14 of these 56 the patients receiving arthroplasty during follow-up and in 118 of the 540 patients who did not.

Hence, there are episodes of deterioration of pain/function in patients both with and without arthroplasty. However, patients without a prosthesis had more time to develop deterioration than patients who received an arthroplasty (median follow-up time 9.0 years [IQR 9.0–10.0] versus 4.5 years [IQR 3.3–6.3], p < 0.001). When corrected for follow-up duration, arthroplasty patients had higher odds on a first deterioration in pain (odds ratio 3.2 [95%CI 1.4–7.2]) and function (odds ratio 2.3 [CI 1.1–5.0]) than patients without arthroplasty.

### Course of pain/function and timing of arthroplasty

**Figure 3.**
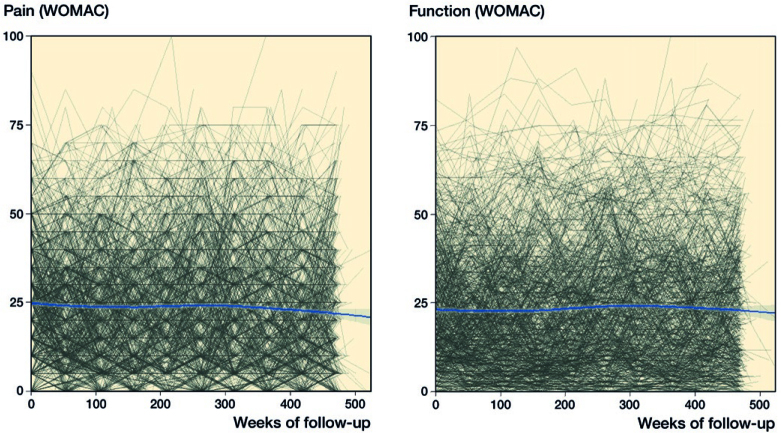
Spaghetti plots of the course of pain and function, (left) individual WOMAC pain scores, and (right) individual WOMAC function scores. The blue lines represent the mean WOMAC score.

Individual pain and function trajectories are depicted in Figure 3. Patients had some variation in pain/function levels over time (deteriorations were often followed by improvements), but overall pain and function seemed to be stable during follow-up as depicted by the plotted line. In accordance with this, our adjusted mixed models showed stable pain levels over time, –0.1 (CI –0.2 to 0.1) points/year (Table 3), which is –0.5 points/decade (CI –2.1 to 0.5). Function significantly deteriorated over time by 0.3 (CI 0.2–0.4) points/year on a 100-point scale (Table 3), which is 2.6 points/decade (CI 1.6–4.2).

The estimates from the joint model showed that higher levels on WOMAC score of pain and function significantly increased the hazard of receiving THA/TKA (1.08 [CI 1.06–1.10] for pain and 1.07 [CI 1.05–1.08] for function) (Table 4).

**Table 4.** Effect estimates of adjusted joint models for total hip and knee arthroplasty

Factor	Estimates for pain HR (95% CI)	Estimates for function HR (95% CI)
Pain	1.08 (1.06–1.10)	
Function	1.07 (1.05–1.08)	
Sex^a^	0.61 (0.35–1.07)	0.76 (0.44: 1.31)
Age (years)	1.06 (1.01–1.10)	1.05 (1.01–1.10)
BMI	0.94 (0.89–0.99)	0.94 (0.90–1.00)
Maximal Kellgren and Lawrence score	2.96 (1.55–5.67)	2.95 (1.54–5.64)
Number of painful joints	0.73 (0.57–0.93)	0.72 (0.56–0.92)
Comorbidities	0.77 (0.66–0.91)	0.79 (0.68–0.92)

HR = hazard ratio.

a Men as reference category

**Table 3.** Fixed effects of adjusted linear mixed-effects models describing the course of pain and function over time

Factor	Estimates for pain Beta (95% CI)	Estimates for function Beta (95% CI)
Time (years)	–0.1 (–0.2 to 0.1)	0.3 (0.2 to 0.4)
Sex ^a^	3.8 (1.5 to 6.0)	2.6 (0.3 to 4.9)
Age (years)	0.0 (–0.2 to 0.2)	0.1 (–0.1 to 0.3)
BMI	0.8 (0.6 to 1.0)	0.9 (0.7 to 1.1)
Maximal Kellgren and Lawrence score	1.0 (–0.9 to 2.8)	1.6 (–0.4 to 3.5)
Number of painful joints	2.1 (1.2 to 3.0)	2.4 (1.5 to 3.4)
Comorbidities	2.5 (1.9 to 3.1)	2.8 (2.1 to 3.4)

**^a^**Men as reference category

### Sensitivity analysis

Adjusting for maximal K&L during follow-up did not change our results (data not shown). When investigating the effect of the long-term changes of pain and function over time by adding the slope of the initial mixed models, the effect estimates of pain and function levels on receiving THA/TKA did not change (Table 5, see Supplementary data), nor did the slope itself increase the risk of receiving THA/TKA. The wide 95% CIs show that the model could not properly estimate the effect of the slope. Hence, long-term changes in pain or function over time did not affect the risk of receiving THA/TKA, when adjusted for other covariates and the level of pain/function.

**Table 5.** Sensitivity analyses

	HR (95% CI) adjusted for maximal K&L at baseline	HR (95% CI) corrected for maximal K&L during follow-up
**Joint Model with slope included for pain**		
Pain	1.08 (1.06–1.10)	1.07 (1.05–1.09)
Pain slope	225 (0–276 × 10^6^)	2.46 (0–15 × 10^6^)
Sex**^a^**	0.60 (0.34–1.05)	0.68 (0.39–1.20)
Age (years)	1.06 (1.01–1.10)	1.05 (1.01–1.10)
BMI	0.94 (0.89–0.99)	0.95 (0.90–1.00)
Maximal K&L score	2.93 (1.53–5.61)	1.85 (1.38–2.47)
Number of painful joints	0.73 (0.57–0.93)	0.76 (0.60–0.98)
Comorbidities	0.78 (0.67– 0.92)	0.79 (0.67–0.93)
**Joint Model with slope included for function**		
Function	1.06 (1.05–1.08)	1.06 (1.04–1.07)
Function slope	1,801 (0–683 × 10^6^)	33.5 (0–52 × 10^6^)
Sex**^a^**	0.75 (0.43–1.30)	0.81 (0.46–1.40)
Age (years)	1.05 (1.01: 1.10)	1.05 (1.00: 1.10)
BMI	0.95 (0.90–1.00)	0.95 (0.90–1.00)
Maximal K&L score	2.95 (1.54–5.64)	1.85 (1.39–2.46)
Number of painful joints	0.73 (0.57–0.94)	0.78 (0.61–1.00)
Comorbidities	0.81 (0.69–0.94)	0.82 (0.71–0.96)

HR: hazard ratio.

K&L: Kellgren and Lawrence

a Men as reference category

**Table 2.** Baseline characteristics of our study population versus the baseline characteristics of the excluded patients/non-responders

	Current study population n = 868	Excluded patients/ non-responders n = 134	p-value
Age	56 (5.2)	56 (5.3)	0.6
Sex:			
Male	179 (21)	31 (23)	
Female	689 (79)	103 (77)	0.5
BMI	26 (4.1)	26 (3.6)	0.3
Comorbidities	1.9 (1.5)	1.9 (1.8)	0.7
Number of painful joints:			
1	262 (30)	46 (34)	
2	370 (43)	55 (41)	
3	125 (14)	19 (14)	
4	111 (13)	14 (10)	0.8
Maximal Kellgren and Lawrence score:			
0	302 (35)	10 (8)	
1	566 (65)	10 (8)	0.2
WOMAC subscales			
Pain	25 (17)	29 (19)	0.01
Function	23 (17)	27 (20)	0.01
Stiffness	33 (21)	36 (23)	0.1

WOMAC; Western Ontario and McMaster Universities Osteoarthritis Index. Continuous variables are shown as mean (SD), categorical variables are shown as number (percentage)

## Discussion

In this cohort of patients with early OA symptoms at inclusion, we investigated whether pain and function changes were associated with receiving THA/TKA. During 9 years of follow-up one-tenth of the patients received an arthroplasty. Approximately two-thirds of all patients showed at least 1 episode of deterioration of pain or function during follow-up. In about one-fifth these deteriorations were followed by another deterioration in the following year. At group level, pain and function remained fairly stable over time. We showed that higher pain and function levels were associated with an increased risk of receiving THA/TKA. Adding the individual long-term changes in pain or function over time did not affect the risk of receiving THA/TKA. Thus, it seems that pain and function levels rather than long-term changes are associated with timing of THA/TKA.

At group level, we showed fairly stable levels of pain and function over 9 years of follow-up, suggesting that major debilitating variables in OA, like pain/functional loss, are persistent rather than worsening. This is in accordance with other studies. A study from the Osteoarthritis Initiative identified 5 relatively stable pain trajectories over 6 years of time in knee OA patients (Collins et al. 2014). These trajectories differed in severity, but all remained stable during follow-up and none showed considerable deterioration/improvement. Recently, in the same cohort White et al. (2016) showed 5 different trajectories of function over a period of 7 years. Overall, function remained stable, although a subgroup of 5% of the cohort showed progressive deterioration. However, the mean deterioration was only 13 out of 68 points on the WOMAC scale. A different study identified 4 trajectories in total WOMAC score in hip/knee OA: increasing scores (18% of the cohort), stable (40%), decreasing (24%), and unstable trajectories (18%) (Leffondre et al. 2004). Hence, for most patients, trajectories remained fairly stable in these studies, although in subgroups of patients improvement and deterioration were present.

The only study investigating trajectories of pain and function in arthroplasty patients showed that patients tend to worsen in function and pain in the last 2 years before arthroplasty (Riddle et al. 2013). However, they did not compare pain/function trajectories with patients not receiving arthroplasty. Our study showed that the long-term change in pain and function over time was not associated with arthroplasty, and thus not different from those not receiving arthroplasty, whereas levels of pain and function were. As patients receiving an arthroplasty reported worse function and more pain at baseline, the levels at which a patient presents him-/herself at first visit for OA complaints seem to determine mostly the risk of arthroplasty. This conclusion is strengthened by the finding that approximately 80% of the arthroplasties were conducted within the first 5 years. Moreover we assembled all patients at inception of symptoms, implying a rather similar disease stage among all patients. As patients receiving THA/TKA showed more complaints at baseline than patients not receiving a prosthesis, these patients may represent a different patient group. One should try to identify this group when commencing clinical care, so that early non-operative treatment can be better targeted.

However, besides modelling the natural course of OA, we also modelled surgeons’ and patients’ behaviour as they decide together that arthroplasty is warranted. If a patient is eager to have an arthroplasty he/she has a higher chance of receiving it than when the same patient is reluctant. Moreover, some surgeons will advise arthroplasty sooner than others. These variations might be amplified by the absence of clear indication criteria for THA/TKA (Ackerman et al. 2009, Dieppe et al. 2009, Gademan et al. 2016, Skou et al. 2016, Riddle and Perera 2017). As patients receiving arthroplasty may not have been the only ones needing arthroplasty, these variations may have diluted our analysis, leading to either an under- or an overestimation of our effect sizes. Nonetheless, by modelling clinical practice, our estimates represent real-life effect sizes.

Concerning optimal timing of arthroplasty, one could speculate that it is beneficial to postpone surgery when possible to reduce the risk of revision surgery. Patients with better preoperative function attain better postoperative functional levels than patients with worse preoperative function (Hofstede et al. 2016) but as we showed that patients seem to remain fairly stable over time, then lowering the risk of revision surgery by postponing the primary surgery might outweigh the risk of fast deterioration from a lifetime perspective. Nonetheless, no conclusions about timing can be based on this single study with early OA patients at baseline. Our results first need to be validated in other OA cohorts.

Limitations of our study include that we had no information on pharmacological or other non-operative treatment. Different treatment strategies might lead to differences in pain/function trajectories. However, as THA/TKA is the end-stage intervention, both surgeon and patient were convinced that THA/TKA was the ultimate treatment option. Furthermore, patients were treated according to the Dutch OA guidelines. Therefore we expect this to have only small effects on our results. Second, patients had early OA symptoms at baseline and therefore represent a different patient group than those seen by an orthopedic surgeon. This could make our results less generalizable to orthopedic practice. However, by the time the patients received arthroplasty, they were seen by an orthopedic surgeon. Moreover, an advantage of this population is that we could assess the course of pain/function from first complaints onwards, giving a complete view of the course of OA complaints. Moreover, by including all patients at inception of their complaints, selection bias was avoided. Nonetheless our results should be validated in another OA population. Third, our analysis concerns actual THA/TKA decisions, which may not be indicative of optimal THA/TKA decision-making. Fourth, often knee and hip OA are reported separately, but in the current study we could not make a such a distinction as patients often had both hip and knee complaints. Finally, we are aware that adjusting for maximal K&L during follow-up is not a statistically preferred method (maximal K&L should have been added as a time-varying covariate). However, this method would complicate our analysis and the interpretation of our results. Furthermore, adjusting for the maximal K&L most likely reflects the maximum effect of adjustment.

In summary, pain and function levels rather than long-term pain and function changes over time (which can be seen as the progression rate) are associated with timing of THA/TKA in early OA patients.

## Supplementary data

Tables 2 and 5, and Figures 1–2 are available as supplementary data in the online version of this article, http://dx.doi.org/

## Supplementary data

## Supplementary Material

Supplemental Material
