# Supplementary material for: The course of pain and function in osteoarthritis and timing of arthroplasty: the CHECK cohort
Source: Acta Orthop. 2018 Oct 23;89(5):528–34. doi: 10.1080/17453674.2018.1502533 (PMC6202767; doi:10.1080/17453674.2018.1502533)
Supplement: Supplemental Material [file IORT_A_1502533_SM4829.pdf]

Supplementary data

Table 2. Baseline characteristics of our study population versus the baseline characteristics of the excluded patients/non-responders

	Current study population n = 868	Excluded patients/ non-responders n = 134	p-value
Age	56 (5.2)	56 (5.3)	0.6
Sex:			
Male	179 (21)	31 (23)	
Female	689 (79)	103 (77)	0.5
BMI	26 (4.1)	26 (3.6)	0.3
Comorbidities	1.9 (1.5)	1.9 (1.8)	0.7
Number of painful joints:			
1	262 (30)	46 (34)	
2	370 (43)	55 (41)	
3	125 (14)	19 (14)	
4	111 (13)	14 (10)	0.8
Maximal Kellgren and Lawrence score:			
0	302 (35)	10 (8)	
1	566 (65)	10 (8)	0.2
WOMAC subscales			
Pain	25 (17)	29 (19)	0.01
Function	23 (17)	27 (20)	0.01
Stiffness	33 (21)	36 (23)	0.1

WOMAC; Western Ontario and McMaster Universities Osteoarthritis Index. Continuous variables are shown as mean (SD), categorical variables are shown as number (percentage)

Table 5. Sensitivity analyses

	HR (95% CI) adjusted for maximal K&L at baseline	HR (95% CI) corrected for maximal K&L during follow-up
**Joint Model with slope included for pain**		
Pain	1.08 (1.06–1.10)	1.07 (1.05–1.09)
Pain slope	225 (0–276×10^6)	2.46 (0–15×10^6)
Sex [a]	0.60 (0.34–1.05)	0.68 (0.39–1.20)
Age (years)	1.06 (1.01–1.10)	1.05 (1.01–1.10)
BMI	0.94 (0.89–0.99)	0.95 (0.90–1.00)
Maximal K&L score	2.93 (1.53–5.61)	1.85 (1.38–2.47)
Number of painful joints	0.73 (0.57–0.93)	0.76 (0.60–0.98)
Comorbidities	0.78 (0.67– 0.92)	0.79 (0.67–0.93)
**Joint Model with slope included for function**		
Function	1.06 (1.05–1.08)	1.06 (1.04–1.07)
Function slope	1,801 (0–683×10^6)	33.5 (0–52×10^6)
Sex [a]	0.75 (0.43–1.30)	0.81 (0.46–1.40)
Age (years)	1.05 (1.01: 1.10)	1.05 (1.00: 1.10)
BMI	0.95 (0.90–1.00)	0.95 (0.90–1.00)
Maximal K&L score	2.95 (1.54–5.64)	1.85 (1.39–2.46)
Number of painful joints	0.73 (0.57–0.94)	0.78 (0.61–1.00)
Comorbidities	0.81 (0.69–0.94)	0.82 (0.71–0.96)

HR: hazard ratio.
K&L: Kellgren and Lawrence
[a] Men as reference category

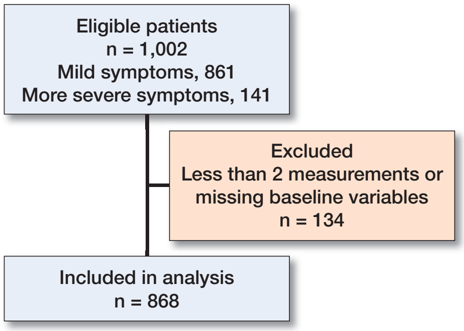

Figure 1. Flow chart.

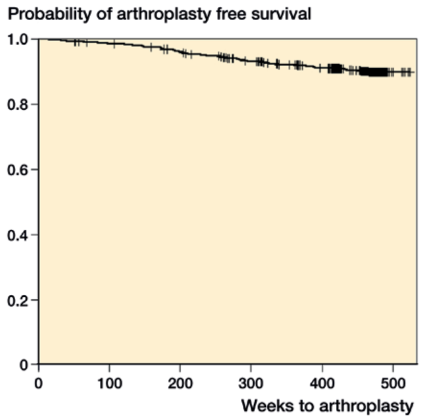

Figure 2. Kaplan–Meier curve for arthroplasty free survival. Censoring is symbolized by "+".

 Acta Orthopaedica 2018; 89 (DOI: 10.1080/17453674.2018.1502533)

SUPPLEMENTARY FILE

R-code mixed effects model and joint model.

#Mixed model pain:
```
fitlmecov <- lme(womacpain~timetofollowup+gender+age+bmi+max_kl+painfulljoints+comorbidities, random=~timetofollowup |patientid,
data=d2, na.action=na.omit)
```

#Mixed model function:
```
fitlmecov2 <- lme(womacfunction~timetofollowup +gender+age+bmi+max_kl+painfulljoints+comorbidities, random=~timetofollowup |patientid,
data=d2, na.action=na.omit)
```

#Cox regression:
```
coxFit <- coxph(Surv(timetoevent, arthroplasy) ~ gender + age +bmi+max_kl+painfulljoints+comorbidities, data = ds2, x = TRUE)
```

#Joint Model pain:
```
fit.JM <- jointModel(fitlmecov, coxFit, timeVar=”timetofollowup”, method=”piecewise-PH-aGH”, verbose=TRUE)
summary(fit.JM)
confint(fit.JM, parm = “Event”)
round(exp(confint(fit.JM, parm = “Event”)), 2)
```

#Joint Model pain + slope:
```
dForm <- list(fixed = ~ 1,
        indFixed = 2,
        random = ~ 1,
        indRandom = 2)
fit.JM.slope <- jointModel(fitlmecov, coxFit,
            timeVar=”timetofollowup”,
            method=”piecewise-PH-aGH”,
            parameterization = “slope”,
            derivForm = dForm,
            verbose=TRUE)
summary(fit.JM.slope)

fit.JM.both <- jointModel(fitlmecov, coxFit,
              timeVar=”timetofollowup”,
              method=”piecewise-PH-aGH”,
              parameterization = “both”,
              derivForm = dForm,
              verbose=TRUE,
             control = list(GHk = 9))
summary(fit.JM.both)
confint(fit.JM.both, parm = “Event”)
round(exp(confint(fit.JM.both, parm = “Event”)), 2)
```

#Joint Model function:
```
fit.JM2 <- jointModel(fitlmecov2, coxFit, timeVar=”timetofollowup”, method=”piecewise-PH-aGH”, verbose=TRUE)
summary(fit.JM2)
confint(fit.JM2, parm = “Event”)
round(exp(confint(fit.JM2, parm = “Event”)), 2)
```

#Joint Model function + slope:
```
fit.JM.slope2 <- jointModel(fitlmecov2, coxFit,
              timeVar=”timetofollowup”,
              method=”piecewise-PH-aGH”,
              parameterization = “slope”,
              derivForm = dForm,
              verbose=TRUE)
summary(fit.JM.slope2)

fit.JM.both2 <- jointModel(fitlmecov2, coxFit,
             timeVar=”timetofollowup”,
             method=”piecewise-PH-aGH”,
             parameterization = “both”,
             derivForm = dForm,
             verbose=TRUE,
             control = list(GHk = 9))
summary(fit.JM.both2)
confint(fit.JM.both2, parm = “Event”)
round(exp(confint(fit.JM.both2, parm = “Event”)), 2)
```
